# Thermoregulatory responses during road races in hot-humid conditions at the 2019 Athletics World Championships

**DOI:** 10.1152/japplphysiol.00348.2022

**Published:** 2023-04-06

**Authors:** Polly Aylwin, George Havenith, Marco Cardinale, Alexander Lloyd, Mohammed Ihsan, Lee Taylor, Paolo Emilio Adami, Marine Alhammoud, Juan-Manuel Alonso, Nicolas Bouscaren, Sebastian Buitrago, Christopher Esh, Josu Gomez-Ezeiza, Frederic Garrandes, Mariem Labidi, Gűnter Lange, Sébastien Moussay, Khouloud Mtibaa, Nathan Townsend, Mathew Wilson, Stéphane Bermon, Sebastien Racinais

**Affiliations:** ^1^Environmental Ergonomics Research Centre, Loughborough University, Loughborough, United Kingdom; ^2^Aspetar Orthopaedic and Sports Medicine Hospital, Doha, Qatar; ^3^Institute of Sport Exercise and Health (ISEH), University College London, London, United Kingdom; ^4^Human Potential Translational Research Program, Yong Loo Lin School of Medicine, National University of Singapore, Singapore; ^5^School of Sport, Exercise and Health Sciences, Loughborough University, Loughborough, United Kingdom; ^6^Human Performance Research Centre, University of Technology Sydney (UTS), Sydney, New South Wales, Australia; ^7^Health and Science Department, World Athletics, Monaco, Principality of Monaco; ^8^Inserm CIC1410, CHU Réunion, La Réunion, France; ^9^Univ Lyon, UJM-Saint-Etienne, Inter-university Laboratory of Human Movement Biology, Saint-Etienne, France; ^10^Olympic Training and Service Centre Lower Saxony, Hannover, Germany; ^11^Division of Orthopaedic Surgery, Faculty of Medicine and Health Sciences, Institute of Sport and Exercise Medicine, Stellenbosch University, Stellenbosch, South Africa; ^12^Unicaen, Inserm, Comete, GIP Cyceron, Normandie Université, Caen, France; ^13^College of Health and Life Sciences, Hamad Bin Khalifa University, Doha, Qatar; ^14^LAMHESS, Université Côte d’Azur, Nice, France

**Keywords:** competition, endurance, hot temperatures, thermography, thermoregulation

## Abstract

The purpose of this study was to characterize thermoregulatory and performance responses of elite road-race athletes, while competing in hot, humid, night-time conditions during the 2019 IAAF World Athletic Championships. Male and female athletes, competing in the 20 km racewalk (*n* = 20 males, 24 females), 50 km racewalk (*n* = 19 males, 8 females), and marathon (*n* = 15 males, 22 females) participated. Exposed mean skin (T_sk_) and continuous core body (T_c_) temperature were recorded with infrared thermography and ingestible telemetry pill, respectively. The range of ambient conditions (recorded roadside) was 29.3°C–32.7°C air temperature, 46%–81% relative humidity, 0.1–1.7 m·s^−1^ air velocity, and 23.5°C–30.6°C wet bulb globe temperature. T_c_ increased by 1.5 ± 0.1°C but mean T_sk_ decreased by 1.5 ± 0.4°C over the duration of the races. T_sk_ and T_c_ changed most rapidly at the start of the races and then plateaued, with T_c_ showing a rapid increase again at the end, in a pattern mirroring pacing. Performance times were between 3% and 20% (mean = 113 ± 6%) longer during the championships compared with the personal best (PB) of athletes. Overall mean performance relative to PB was correlated with the wet-bulb globe temperature (WBGT) of each race (*R*^2^ = 0.89), but not with thermophysiological variables (*R*^2^ ≤ 0.3). As previously reported in exercise heat stress, in this field study T_c_ rose with exercise duration, whereas T_sk_ showed a decline. The latter contradicts the commonly recorded rise and plateau in laboratory studies at similar ambient temperatures but without realistic air movement.

**NEW & NOTEWORTHY** This paper provides a kinetic observation of both core and skin temperatures in 108 elite athletes, during various outdoor competition events, adding to the very limited data so far available in the literature taken during elite competitions. The field skin temperature findings contrast previous laboratory findings, likely due to differences in relative air velocity and its impact on the evaporation of sweat. The rapid rise in skin temperature following cessation of exercise highlights the importance of infrared thermography measurements being taken during motion, not during breaks, when being used as a measurement of skin temperature during exercise.

## Graphical abstract

**Figure:**
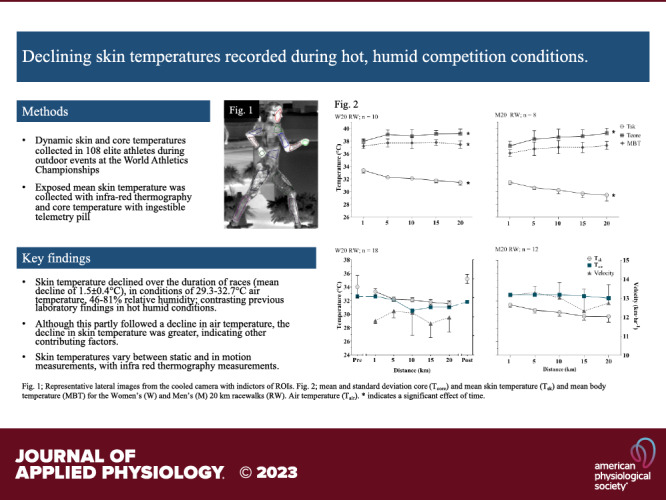

## INTRODUCTION

Climate change imposes an ever-increasing global surface temperature and incidence of extreme weather events, including heat waves. One of the greatest impacts of climate change to humans is increased morbidity and mortality inflicted by elevated environmental conditions (1). Sporting events are not only threatened by heat waves, but increasingly taking place in known hot conditions, such as the 2020 Tokyo Summer Olympics or the annual Tennis Australian Open. Due to the increasing number of events in hot conditions, sporting organizations are under greater pressure to implement appropriate protection. For example, at the 2019 IAAF World Athletics Championships the track and field events were held in an air-conditioned stadium, whereas the road races (marathon and race-walks) were held in the middle of the night, to avoid solar load. Effective protection can only be established if we fully understand the thermophysiological burden athletes are under.

The thermoregulatory mechanisms responsible for degraded performance have been extensively studied; the methods utilized are numerous, but not without limitations. Importantly, research conducted in the laboratory has some fundamental differences to field-based research. For example, treadmill or cycle ergometer exercise is static and although fans are often used to simulate the airflow that is created by movement velocity, this is infrequently matched in speed, nor is it realistically distributed across the body and the turbulence intensity is much higher that found outdoors. Thus, the effect this has on evaporative and convective heat transfer is likely underestimated (2). Laboratory testing is also commonly undertaken with recreational or mid-level rather than internationally competing athletes and without real-world factors such as radiation loads from the road surface or environmental or direct solar load, nor the impacts of competition on psychological state and performance. However, the development of noncontact, noninvasive measures, such as ingestible telemetry pills for core temperature (T_c_) and wireless iButtons, both with data-logging capabilities, or infrared thermography (IRT) for mean skin temperature (T_sk_) has increased the ability to measure thermoregulatory responses in elite athletes during competition (3–8).

Recent research investigating thermoregulatory responses to competitive sport includes cycling championships (4, 9) and Tokyo 2020 test events (10); with Racinais et al. (4) recording T_c_ up to 41.5°C during competition in elite cyclists. Two recent papers have also sought to characterize T_c_ (7) and T_sk_ (6) during racewalking and running, respectively. Stevens et al. (7) reported a rise and plateau of T_c_, during a 20 km racewalk, in 25°C and 74% relative humidity, with no air velocity reported, however, this was in only two participants and did not report skin temperature. Ganse and Degens (6) investigated master athletes with IRT during a 10 km road race, in 22°C, 88% relative humidity, and 8 m·s^−1^ wind speed, but reported T_sk_ values (up to 41°C) beyond those traditionally reported in human exercise studies and hence data should be taken with caution. The application of IRT in their study had several limitations (11), including the use of a FLIR ONE Pro camera with low accuracy and low resolution, and images highly affected by the athlete’s movements (8). The low resolution is particularly problematic as the regions of interest (ROI) were defined as single pixels. To the best of our knowledge, no study has investigated the T_sk_ and T_c_ responses together in elite athletes during competitive running or racewalking. Understanding this phenomena would provide the basis from which policies for protection from heat illness can be informed or the selection of strategies to prevent degradations in performance under heat stress.

Therefore, this study set out to investigate both T_sk_ and T_c_ responses in elite athletes competing in outdoor, hot-humid conditions at the 2019 IAAF World Athletics Championships, Doha. The main aim of the present research is therefore to provide a novel insight into thermoregulatory responses of elite athletes, with true competition data, over and above what has typically been collected in laboratory studies. The secondary aim was to investigate the relationship between performance and the magnitude of thermoregulatory response (T_sk_ and T_c_).

## MATERIALS AND METHODS

### Study Design

This was a cross-sectional, observational study of a large international cohort (*n* = 108) of athletes competing in the male and female 20 km racewalks, 50 km racewalks, and marathons at the 2019 IAAF World Athletics Championships, Doha. Some temperature data collected by the research team at the 2019 Doha IAAF World Athletics Championships have been published earlier (8, 12). The first paper (8) focused purely on the technical side and methodology of dynamic field capturing of skin temperatures with infrared technology rather than the physiology, using the data of the 20k racewalk only. The second paper (12) focused on peak T_c_ and finishing T_c_ and T_sk_ only, without differentiation between races. The present paper looks in detail at more extensive physiological data, separated by individual race and collected and presented across the race duration, and considers this in relation to differing heat stress levels in the individual races.

In this study, the participant numbers vary by race and variable and are included within each figure. All road races took place on a 1–7 km loop (20 km racewalk 1 km; 50 km racewalk 2 km; Marathon 7 km), between the hours of 23:30–05:00, during darkness. The race circuit was level and followed the seashore at “Al Corniche” in Doha, Qatar. Air temperature, relative humidity, air velocity, and wet bulb-globe temperature (WBGT) were measured every 30 min throughout the race with a heat stress meter (Kestrel 4400, Boothwyn, PA) mounted on a tripod with a wind vane, at a height of ∼1.5 m, located ∼4 m from the finish line.

The project was approved by the Anti-Doping Laboratory Qatar ethics committee (E2019000302). All procedures complied with the Declaration of Helsinki and written informed consent was obtained.

### Measurements

#### Body core temperature.

T_c_ was recorded every 30 s by e-Celsius ingestible capsules (BodyCap, Caen, France). Capsules were distributed to athletes on the morning of the race with instructions to consume the pill 4–6 h before the race start. Core data was excluded where it dropped below 36°C.

#### Static exposed skin temperature.

Pre- and postrace static IRT thermograms were obtained [FLIR T600, FLIR Systems Ltd, West Malling, Kent, UK; 480 × 360 focal plane array; Noise Equivalent Temperature (NETD) <0.04°C at 30°C; accuracy ±2°C; accuracy was improved post hoc using reference temperature plates in the image], during the women’s 20 km racewalk, women’s and men’s marathon and post only for the men’s 50 km racewalk. These images were taken at a fixed location 4 m from the camera. Prerace images were taken within 30 min before the race and postrace images as soon as possible, within 50 m of the finish line, before athletes entering the mixed zone.

#### In-race dynamic skin temperature.

The method for measuring Tsk has been evaluated in detail by Aylwin et al. (8), who undertook a comprehensive analysis of the implementation of IRT in the field; this included the impact of motion, camera properties, exposed body surface area, and influence of variability in factors such as the distance of athletes from the camera. In brief, two IRT cameras were positioned trackside with black body and reflective temperature reference plates in sight to improve accuracy above the manufacturer’s specifications. The primary camera used for analysis was a medium wave, cooled indium antimonide camera with an exposure time below 1 ms [medium-wave infrared camera (MWIR): FLIR A6750sc MWIR, FLIR Systems; 640 × 512 Focal Plane Array; 3–5 µm spectral range; 20 mK NETD; max 125 Hz Sampling rate, set to 15–30 Hz; f/2.5; accuracy ±2°C]. The secondary camera used was a long wave, uncooled microbolometer camera with an exposure time around 30 ms [long-wave infrared camera (LWIR); FLIR T1030sc, FLIR Systems Ltd; 1,024 × 768 Focal Plane Array; 7.5–14 µm spectral range; 20 mK at 30°C NETD; max 30 Hz sampling rate; f/1.15; accuracy ±1°C]. The two cameras were mounted on a U-bend of the track, activated and stabilized for a period of ∼45 min before the start of the race. Video was captured every 1 km of the 20 km racewalk, 2 km of the 50 km racewalk, and 7 km of the marathon. T_sk_ was analyzed at 1, 5, 10, 15, and 20 km of the 20 km racewalk, 1, 5, 15, 25, 35, 45, and 49 km of the 50 km racewalk, and 8, 15, 23, 30, and 37 km of the marathons. Analysis of images took place in FLIR software (FLIR ResearchIR Version 4.40. 11.35), by the same researcher throughout. With inputs of radiant temperature, air temperature, relative humidity, distance to the athlete, and emissivity of the athlete’s skin (0.91 for the MWIR camera and 0.98 for the LWIR). Data from the MWIR camera was used preferentially over the LWIR due to its shorter capture time giving fewer movement artifacts (8). ROIs were predetermined before analysis to include all exposed areas of the anterior, lateral, and posterior aspects of the body, covering as much exposed skin surface area as possible. The ROIs covered only exposed body parts, constituting 64% of the total body surface area. We estimate 31% was covered by clothing. Exposed body surface area was typically greater in females, as they wore cropped vests, compared with males with vests covering the torso and longer shorts (8). The individual body sites analyzed for females and males and weightings for each are shown in the appendix. Mean exposed T_sk_ was calculated based on the observed surface area (with corrections for the different distances between camera and athlete in the different views) of the anterior, lateral, and posterior surfaces, as per *Eq. 1*.

(*1*) $$\begin{array}{c} \text{Mean exposed}\;{\text{T}}_{\text{sk}}=0.32\text{Anterior}+0.38\text{Lateral}+0.30\text{Posterior}\; \end{array}.$$

#### Performance.

Finishing times and positions were taken from official competition results and times normalized to the personal best (PB) of athletes, for athletes that finished the race. PB data was obtained from the official World Athletic database. PB was selected as a reference to separate out the climate effects from the effects of individual “talent” in the race outcomes.

### Data Analysis

Values reported are means ± standard deviation. Mean body temperature (MBT) was calculated as 0.8 × T_c_ added to 0.2 × T_sk_ (13; p. 21). Velocity was calculated as the kilometer distance covered, divided by the time between laps. Rate of change is the difference between the temperatures at each shown lap divided by the number of laps between the two measurements. Analysis of T_sk_ and T_c_ during the race only included individuals who finished the entire race, unless otherwise stated [Racinais et al. (12) provide a breakdown of athletes that did not finish and a physiological comparison with finishers]. The effect of time on T_sk_ was analyzed by repeated measures one-way ANOVA, over five measurements for the 20 km racewalk, seven for the 50 km racewalk, and five measurements for the marathon. A linear mixed model was fitted to analyze the effect of time on T_c_. Analysis was performed with IBM SPSS Statistics 25. The women’s 50 km racewalk and marathon T_c_ was not statistically analyzed due insufficient T_c_ data. The relationship between performance (absolute and relative to PB) and final T_sk_ and T_c_, core-to-skin gradient, and the change in each was analyzed by Pearson’s correlation. The alpha level was set to *P* < 0.05.

## RESULTS

**Figure 1.**
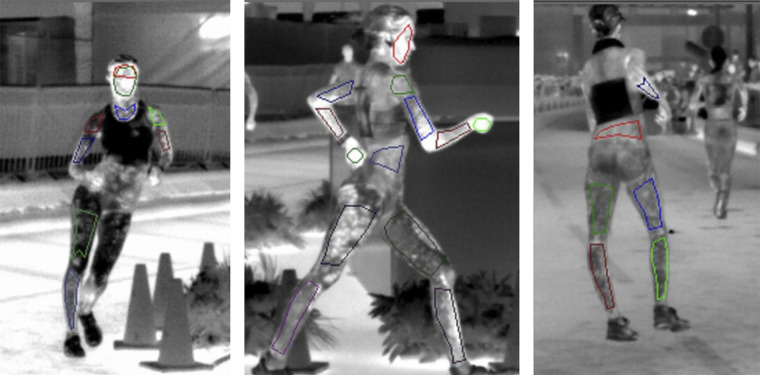
Images taken from the MWIR camera, of female athletes, from the anterior, lateral, and posterior perspectives, with indictors of ROIs. ROIs, regions of interest.

The mean environmental conditions during each race are outlined in Table 1. In addition, participant characteristics and performance data, which was in all but one individual cases degraded from personal best is displayed. Figure 1 shows a representative analysis of images from the anterior, lateral, and posterior perspectives, indicating ROIs.

**Table 1.** Environmental conditions during each race, participant characteristics, and performance outcomes

Event	Air Temperature, °C	Relative Humidity, %	Vapor Pressure, kPa	Air Velocity, m·s^−^1^^	WBGT, °C	Age, yr	Height, cm	Weight, kg	Finish Rank (Range)	Time, Min	Time, %PB
W_20km RW	31.6 ± 0.8	77 ± 3	3.6 ± 0.1	0.3 ± 0.4	28.9 ± 0.5	27.0 ± 6.5	162.3 ± 6.9	50.0 ± 4.2	22 (4–39)	100.2 ± 3.8	113 ± 4
M_20km RW	32.7 ± 0.2	81 ± 1	4.0 ± 0.5	1.7 ± 0.4	30.6 ± 0.3	28.4 ± 3.9	178.2 ± 7.1	65.2 ± 5.9	21 (1–39)	94.7 ± 6.0	120 ± 6
W_50km RW	32.7 ± 0.2	76 ± 3	3.4 ± 0.1	0.4 ± 0.7	28.2 ± 0.9	29.8 ± 5.4	167.0 ± 6.4	54.4 ± 6.3	12 (7–17)	293.9 ± 21.8	113 ± 7
M_50km RW	31.1 ± 0.5	76 ± 3	3.4 ± 0.1	0.4 ± 0.7	28.2 ± 0.9	32.7 ± 7.0	180.7 ± 6.1	67.9 ± 5.8	16 (3–26)	263 ± 12.2	116 ± 4
M_Marathon	32.0 ± 0.7	78 ± 3	3.7 ± 0.1	0.1 ± 0.2	29.6 ± 0.3	30.4 ± 6.2	160.9 ± 5.5	48.6 ± 4.8	17 (1–38)	168.4 ± 11.9	114 ± 5
W_Marathon	32.0 ± 0.7	46 ± 1	1.7 ± 0.4	0.4 ± 0.5	23.5 ± 0.4	31.2 ± 3.4	176.3 ± 8.4	61.6 ± 5.5	35 (4–53)	140.5 ± 5.6	103 ± 3

Data are means ± standard deviation, with the exception of rank which is the mean and the range of finishing positions of the athletes participating. The environmental conditions during each race are given from the start of the race until the final athlete finished. Participant characteristics and performance outcomes are given for all participants. M, men’s; RW, racewalk; W, women’s; WBGT, wet-bulb globe temperature; %PB, percent of personal best time.

**Figure 2.**
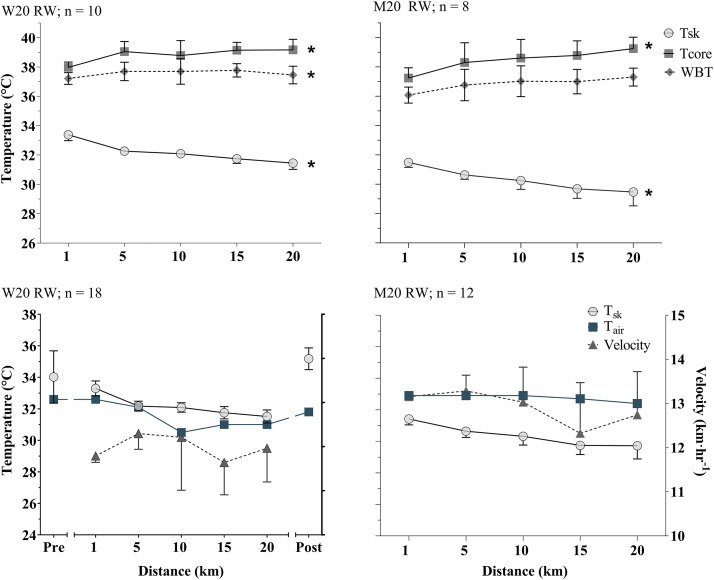
Data are mean ± standard deviation for the Women’s (W) and Men’s (M) 20 km racewalks. *Top* graphs show mean core and skin temperatures and mean body temperature. *Bottom* graphs display mean T_sk_, along with air temperature (T_air_) and the speed of athletes. Note pre and post data were not available for the Men’s race. *Significant effect of time.

**Figure 3.**
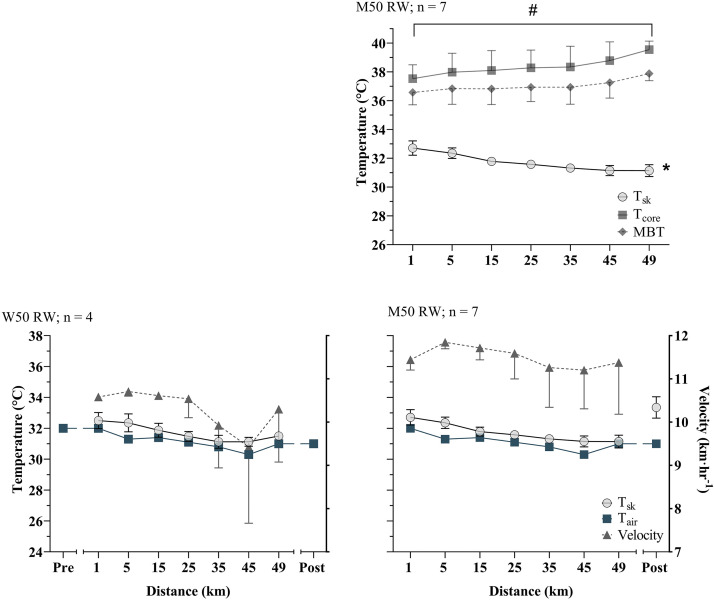
Data are mean ± standard deviation for the Women’s (W) and Men’s (M) 50 km racewalks. *Top* graph shows mean core and skin temperatures. *Bottom* graphs display mean T_sk_, along with air temperature (T_air_) and the speed of athletes. Note there is no core temperature data available for the Women’s race. The final time point in the Women’s race has an *n* = 2 for skin temperature, and pre and post data were not available for the Women’s race or pre for the Men’s. *Significant effect of time, #significant difference between two time points.

**Figure 4.**
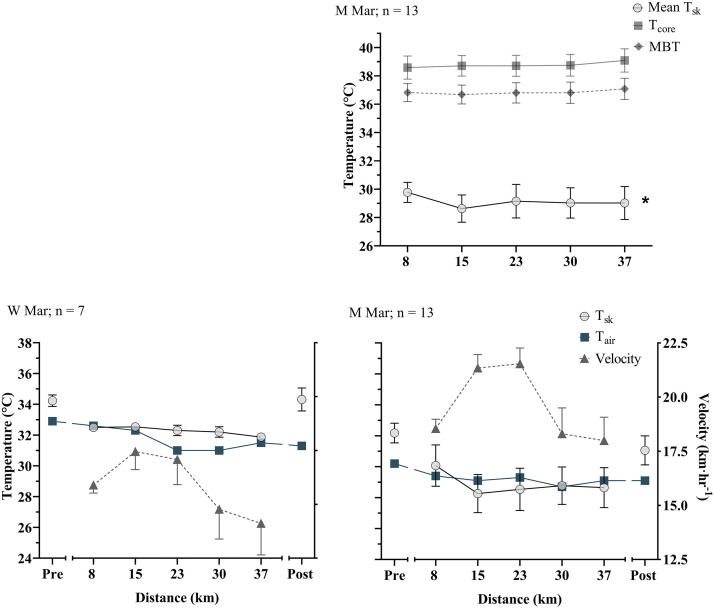
Data are mean ± standard deviation for the Women’s (W) and Men’s (M) marathons. *Top* graph shows mean core and skin temperatures. *Bottom* graphs display mean T_sk_, along with air temperature (T_air_) and the speed of athletes. Note there is no core temperature data available for the Women’s race. The final time point in the Women’s race has an *n* = 2 for skin temperature, and pre and post data were not available for the Women’s race or pre for the Men’s. *Significant effect of time.

**Figure 5.**
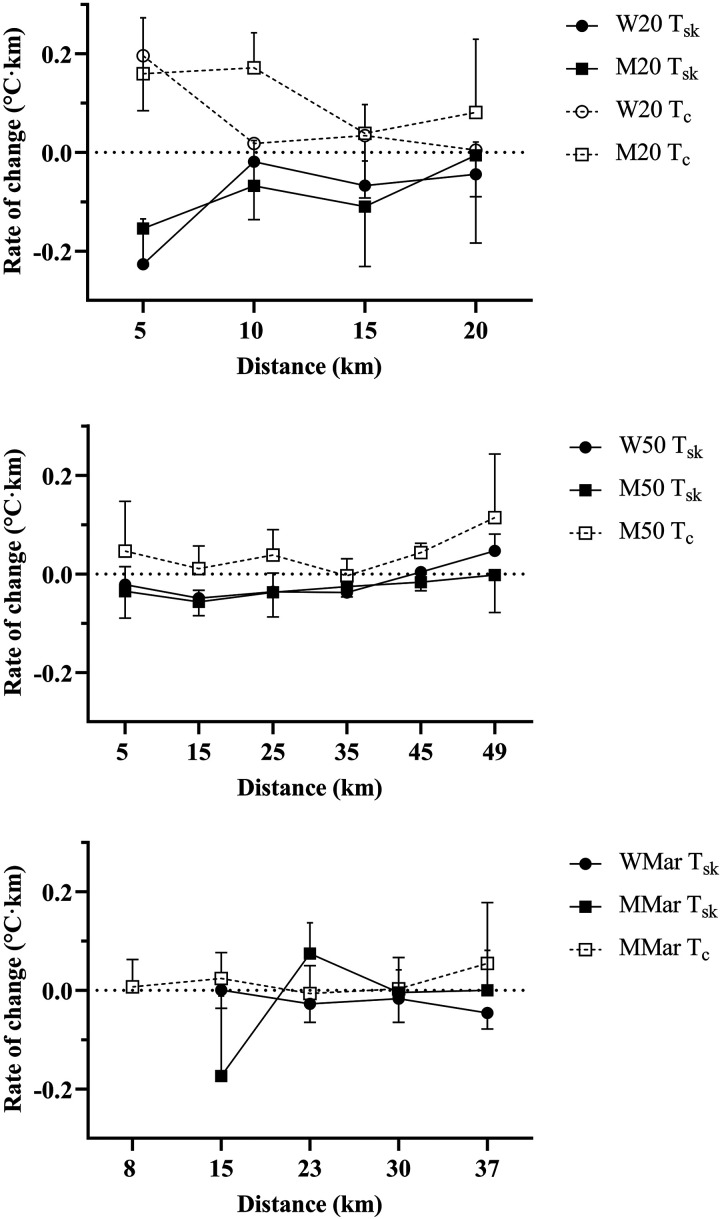
The rate of change per kilometer for skin (solid line) and core (broken line) temperatures for the 20 km racewalk *top*, 50 km racewalk *middle* and marathon, *bottom* graph. Data are expressed as mean ± standard error.

In all events, T_c_ rose but T_sk_ declined over the duration of the race. In all races there was a significant effect of time on T_sk_ (*P* < 0.05), declining between 0.3°C and 2.6°C across events (Figs. 2–4). The pattern of decline in T_sk_ was not linear, typically falling more rapidly at the start of races, slowing during the middle portion of the race, and in some cases showing an increase in the end portion (Fig. 5). Rate of change was, however, more stable for the longer duration marathon and 50 km racewalk than for the 20 km racewalk (Fig. 5). T_sk_ was lower in the men’s 20 km racewalk and marathon than other races, sitting in both below air temperature (Figs. 2 and 4). In the men’s 20 km racewalk T_sk_ was ∼2.0°C lower than air temperature (32.7°C) at below 32°C throughout (Fig. 2) and in the men’s marathon was below the lower air temperature (29.3°C) with T_sk_ ∼29.1°C, falling by 0.7 ± 1.4°C from 29.8 ± 0.7 to 28.9 ± 1.1°C (Fig. 4).

**Figure 6.**
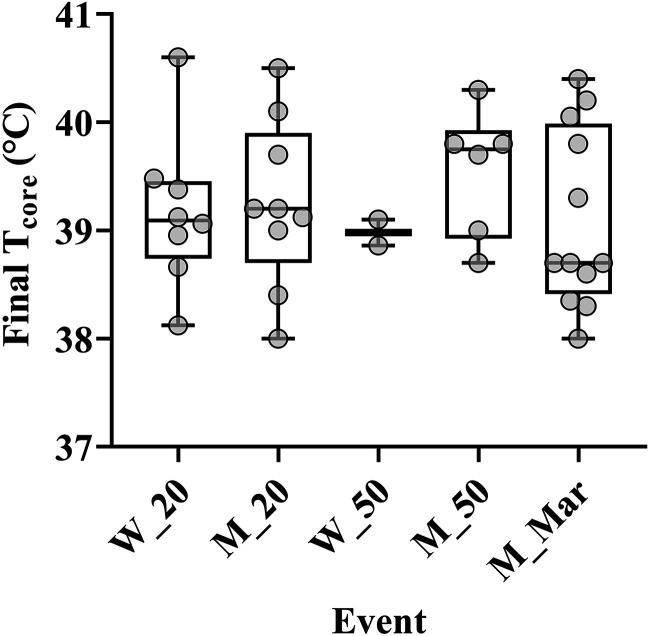
Final core temperature, measured in the last kilometer of each race. Box plot minimum values (error bars).

T_c_ increased between 0.5°C and 2.1°C across events (*P* < 0.05 in the 20 km racewalks and men’s 50 km racewalk; corresponding with the a greater rise in MBT of 0.6°C and 0.7°C, respectively). T_c_ rose most rapidly in the early stages of the races and the rate of increase slowed as the race continued (Fig. 2–4). In the men’s 20 and 50 km racewalks the rate of change in T_c_ showed an increase from the penultimate measurement to the final lap, in a pattern mirroring the increase in the speed of athletes at the end of race, indicative of a sprint finish. These two races also showed an increase in MBT from the start to the end of the race, despite being relatively stable during the race (Fig. 2–4). All participating athletes finished races with T_c_ above 38°C and 16% of athletes finished with T_c_ above 40°C (Fig. 6). The highest individual T_c_ was 40.7°C, recorded during the men’s 20 km racewalk in an athlete that did not finish. The highest from a finishing athlete was 40.6°C during the women’s 20 km racewalk.

### Pre- and Postrace Skin Temperatures

In the races where thermograms were taken of athletes before the start of the race and immediately postrace (women’s 20 km racewalk, women’s and men’s marathon, and post only for the men’s 50 km racewalk), the mean T_sk_ dropped from the prerace value to the first in-race value (mean −1.5 ± 1.5°C, *P* < 0.01) and rose rapidly from the final in-race value to post (mean +2.8 ± 1.1°C, *P* < 0.01; Figs. 2–4). Large increases were seen from the final kilometer to post race of up to 3.7°C, in the women’s 20 km racewalk. Comparing pre to post race mean T_sk_ increased, contrasting the decline that was recorded from the first to last kilometer during the race.

### Performance

The best and the worst mean relative performances were in the men’s marathon at 103 ± 3% and the men’s 20 km racewalk at 120 ± 6% of PB. The relative performance of all races showed a significant correlation with both air temperature (*R*^2^ = 0.85, *y* = 4.5166*x* − 28.203, *P* = 0.0095) and WBGT (*R*^2^ = 0.89, *y* = 2.1534*x* + 52.513, *P* = 0.005), but not with T_sk_ or T_c_ or the gradient between the two (*R*^2^ < 0.3).

### Individual Body Sites

**Figure 7.**
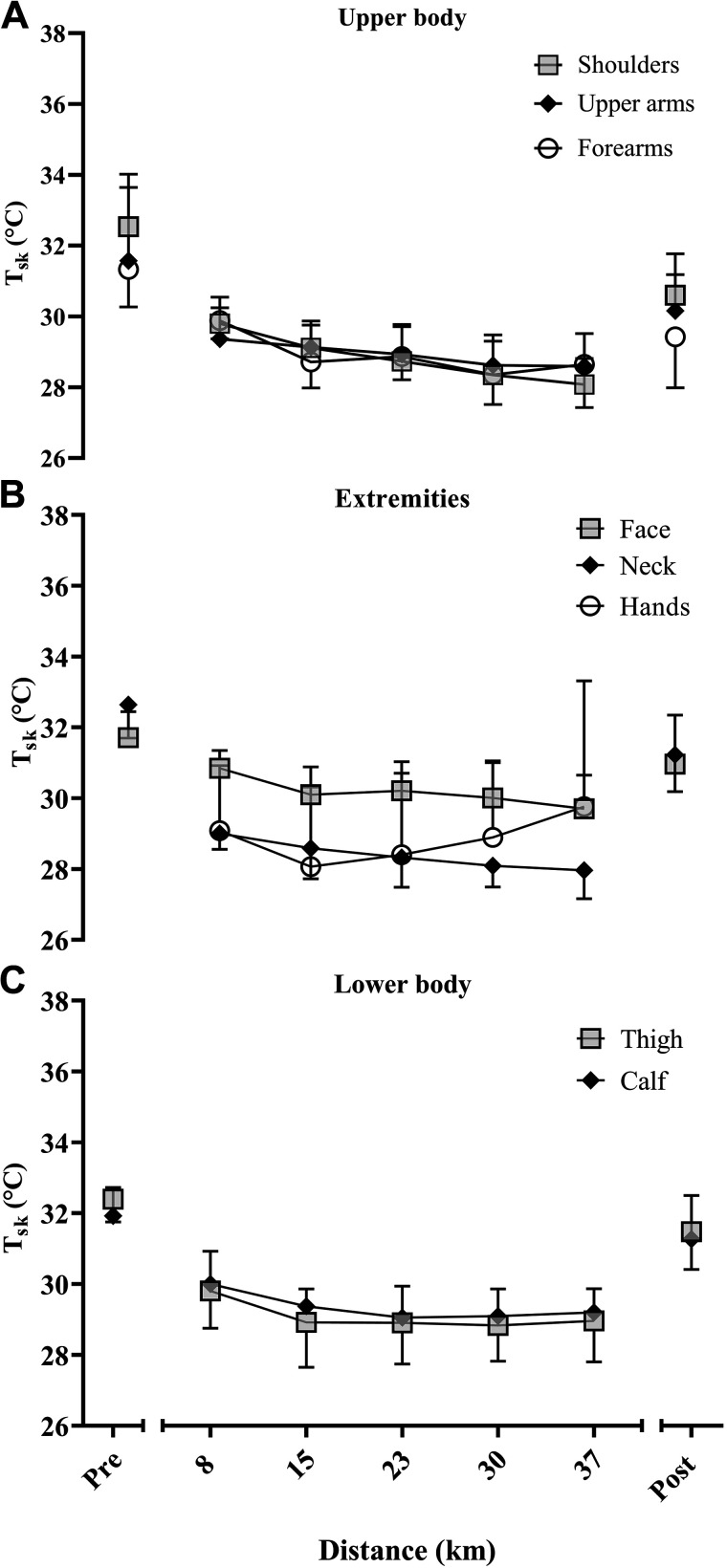
Representative skin temperatures of each ROI, during the men’s marathon, separated by the upper body (*A*; *top* graph), extremities (*B*; *middle* graph), and lower body (*C*; *bottom* graph). Data are mean ± standard deviation. ROIs, regions of interest.

Figure 7 shows the T_sk_ of each ROI, an example has been given for one race (men’s marathon), representative of the pattern of results across races. Overall, the face displayed the highest T_sk_. The hands started with the lowest T_sk_ but increased during races, opposing the typical decrease in mean T_sk_.

## DISCUSSION

We identified a paucity of data on thermoregulatory responses, notably of skin temperature responses, of elite athletes running or race-walking during competition, in hot environments. The competition component as well as the natural climate exposure, create a condition substantially distinct from laboratory studies, and different outcomes may be expected. This paper, therefore, provides a novel insight into the thermoregulatory responses in hot field conditions during actual competition. It is the first large field study (*n* = 108) combining skin, core, and performance responses, over time in multiple road races, during World Championships. Athletes experienced high levels of thermal strain in all events. As expected, T_c_ increased during the race. Contrary to laboratory studies that investigate T_sk_ responses in air temperatures similar to skin, our results showed a declining T_sk_. T_sk_ declined most rapidly early in races and the rate of decrease declined more, the longer the duration of the race. There were no clear relationships between individual participant’s performance and physiological strain indicators, though over all races there was a correlation between race times (% PB) and air temperature (*R*^2^ = 0.85) and WBGT (*R*^2^ = 0.89). However, neither correlated with either mean or final T_sk_.

### Skin Temperature

T_sk_ is a function of the balance between heat transfer from the core to the skin by cutaneous blood flow on one hand and the convective and evaporative heat loss from the skin to the environment on the other (14). In conditions of high heat stress, evaporation of sweat is the primary heat loss mechanism and appears to have been highly effective in this scenario, despite the high humidity and low wind speeds. T_sk_ dropped over the duration of each race, more rapidly in the initial phases and slower as the race progressed (Figs. 2–4). Air temperature also decreased over the duration of races, likely explained by the night-time conditions. This drop in air temperature likely explained a proportion, but not all of the drop in skin temperature. While in the 50 km racewalks T_sk_ was similar to air temperature throughout, in the men’s 20 km racewalk and marathon, although air temperature fell, T_sk_ showed a greater decline. The difference in skin and air temperature was not consistent over the duration of races; which it would have been if the decline in air temperature solely explained the declined in T_sk_. In fact T_sk_ declined more rapidly than air temperature, in most races. Thus, air temperature can only be a partial explanation. The 50 km racewalks may be a closer match to air temperature, as they were the slowest in pace and therefore less affected by the air velocity generated by movement.

The initial rapid decline in T_sk_, was likely due to the increased air speed due to motion and a function of the increasing sweat production and evaporation, which stabilizes after around 20–30 min (15, 16), coinciding with the stabilization of T_sk_. Later on, a slowing pace results in a reduction in air velocity across the skin, reducing the rate of evaporation of sweat and convective heat loss. The speed of athletes changed during races by up to 4 km·h^−1^, which translates into a variation in relative (to the athlete) wind speed of 1 m·s^−1^. Although the absolute wind speeds were low (Table 1; mostly <0.47 m·s^−1^, with only men’s 20 km racewalk at 1.7 m·s^−1^), relative wind speeds and thereby effective evaporation are supported in outdoor exercise by the movement velocity. Therefore, when we compare the data to laboratory studies the pattern of T_sk_ was contrasting. Most laboratory studies show T_sk_ to increase and plateau above pre-exercise levels (17–20). In laboratory studies where exercise is static and air is often still (18) or fan air speeds are below running speed (21) or not applied to the whole body, they may underestimate the evaporative and convective heat loss that occurs in real-world activity. A higher, well distributed over the body, relative air speed in the field (due to the wind and movement combined) is expected to reduce any increase in T_sk_ or, as observed, even reduce it compared with laboratory studies with less realistic winds (22). The impact of relative air movement may be further highlighted by the rapid increase in T_sk_ postrace when athletes became stationary. This has been discussed in a methodology paper by Aylwin et al. (8). This was similarly recorded by Maron et al. (20), who reported a sharp rise and deviation from air temperature, postrace, during a field-based study utilizing thermistors (20). Again, the postrace increase may also be affected by a rapid cutaneous vasodilation, due to cessation of skeletal muscle contraction reducing sympathetic drive and cardiac output demand (23). The stark difference between last lap and post values indicates that one has to be careful when using a protocol of IRT where exercise is briefly interrupted to obtain a static image (8). It should be noted in the present study, although the post picture was taken very close to the finish line (∼50 m), there was limited control over the athlete in the time between crossing the finish line and reaching the testing station whereby, some athletes heavily doused themselves [80% of athletes reported planning on dousing during the race, however, it is not known how many of these doused immediately after finishing (12)]. However, despite such cooling interventions, the observed posttemperature was still higher than the last lap, and therefore any cooling intervention may have reduced, but appears not to have masked this effect.

The lower T_sk_ recorded in comparison with previous laboratory studies, may also be a function of the measurement method and the ability of this method to only measure exposed T_sk_. Although more commonly used, contact techniques also suffer limitations, including interference from the fixation method (24–27), small surface area measurement (28), and lack of feasibility during actual athletic competitions. Guidelines indicate the standardization that should be implemented for accurate and reliable IRT measurements (29–32), which were followed as applicable. However, due to the inability to standardize field testing to the same degree as laboratory studies, before analysis a thorough investigation of the application of IRT to the setting was conducted (8). Although our results appear to produce T_sk_ lower than expected during the race, Merla et al. (33) showed a similar pattern of declining T_sk_, measured with IRT during a graded exercise test. Furthermore, our starting values are similar to those recorded with thermistors, in other studies of similar conditions (34) and therefore, are not a function of the method of measurement used. However, Maughan et al. (33) do not report the clothing coverage over thermistors, a potential contributor to differences in T_sk_ measured by IRT versus contact methods. Clothing creates a microclimate within the layer of insulation next to the skin, which can act as a barrier to heat loss and sweat evaporation (35), thus covered areas are expected to show higher T_sk_, and if we were able to measure the entire body surface area including under clothing, the whole body T_sk_ may be higher. Low T_sk_ may also be a function of the night-time conditions. This omits the effects of solar radiation (36, 37) and introduces a possible influence of circadian rhythm, in comparison with majority of research which takes place in the daytime. However, with the emission of solar radiation, we would still expect T_sk_ to match indoor exercise and it has been previously shown that T_sk_ responses to exercise are not altered by the time of day of the test (38).

The men’s 20 km racewalk and marathon events displayed absolute T_sk_ lower than during other races by ∼2°C. Clothing could be a potential driver for sex differences, however in that case we would expect the men’s T_sk_ to be higher due to higher clothing coverage. A full discussion of the impact of clothing coverage on IRT measurements has been given by Aylwin et al. (8). Metabolic rate could also explain this difference, however in the 50 km racewalk where the men’s and women’s races took place under the same conditions, T_sk_ was similar and T_c_ of men and women in response to the 20 km racewalk were similar, despite different speeds. Therefore, lower T_sk_ was more likely due to the higher air velocity at 1.7 m·s^−1^ in the men’s 20 km racewalk, rather than ≤0.4 m·s^−1^ in other races, compensating for the high vapor pressure (Table 1) and in the men’s marathon due to the lower air temperature (29.3°C vs. 31.7°C for the other races) and vapor pressure (1.7 vs. 3.6 kPa). The water vapor pressure determines the transfer of vapor from the skin’s surface to the surrounding air. As vapor pressure of the air approaches that of the skin, evaporation is hindered (39). With the exception of the men’s 20 km racewalk, with high wind speed, the data showed indeed that the lower the vapor pressure, the lower the T_sk_.

In the men’s 20 km racewalk, the water station was located only 100 m before the position of thermal cameras, and therefore many were dousing just before imaging. The additional external cooling may be a contributing factor in T_sk_ dropping below air temperature; explaining why the lower T_sk_ did not translate into a better performance. Certain individuals displayed a particularly low starting T_sk_, e.g., during the women’s 20 km racewalk one individual had a starting T_sk_ of 28°C; this was deemed not to be an outlier, as images showed what is likely to have been aggressive precooling of the lower body. Interestingly this individual’s T_sk_, did not remain lower than other participants once the race began. They did, however, record the lowest ending T_c_ (unfortunately no starting T_c_ is available for this race). Overall, for those with the lowest starting T_sk_ in each race, this converged with other athletes, typically within half to two-thirds of the way through the race. Only one individual displayed T_sk_ lower than other athletes throughout the whole race.

### Body Core Temperature

In compensable conditions, T_c_ is relatively independent of environmental conditions and instead modulated by workload (19). In all races T_c_ increased over the duration of the race by 1.5 ± 0.4°C, in a pattern mirroring pacing with more rapid increases in T_c_ when pace was quickest (Figs. 2–4) and thus metabolic heat production highest. Furthermore, the rate of change during the 50 km racewalk was slowest, along with the slowest pace (Figs. 3 and 5). The T_c_ values recorded were similar to those in previous studies; for example, in elite 20 km racewalk competitors in moderate conditions (7). However, their study had a small participant number (*n* = 2, in the 20 km racewalk) and did not assess T_sk_. Furthermore, a group of 18 heat-acclimatized male soldiers reached T_c_ of 39°C, and over half reached >40°C, during a 21 km running race, in 27°C air temperature and 87% relative humidity (40) as well as in marathon runners (20). Our findings add to a growing body of evidence, that athletes can continue exercise beyond a T_c_ of 40°C, without exhaustion (4, 7, 20, 40) as those who reached >40°C, did not drop out of races at this point. In a 15 km running event, at 11°C WBGT, 15% of athletes recorded T_c_ above 40°C (41). The similar incidence rate to our athletes (16%) in their moderate conditions is an interesting comparison. None of the athletes in their study recorded any signs of heat illness, nor saw a decline in pacing over the duration of the race. Therefore, supporting that it may not be a core temperature mechanism regulating the decline in performance. Although cutaneous blood flow was likely to be high in their athletes to compensate for the rise in T_c_, effective heat loss from the skin’s surface, due to the moderate conditions means that performance did not need to be degraded to reduce the rate of heat production; opposing our findings due to higher conditions. It is plausible that T_sk_ in their race was much cooler than in the present.

T_c_ achieved does not appear to be the mechanism modulating performance degradation in the heat in this study. No significant correlations between relative performance and individual T_c_ were observed. In addition, during the men’s marathon, which displayed the best relative performance, absolute T_c_ was similar to other races. Thus, a pacing behavior that controls individual T_c_ may be present, as opposed to T_c_ driving a reduction in pacing. The exact mechanism driving the performance of the athletes’ pacing can only be speculatively inferred from the data collected in this study.

### Performance

As expected, aerobic performance was hindered, the magnitude of which correlated with the WBGT during the race (*R*^2^ = 0.89); with only 1 athlete, out of 219 matching their PB. WBGT has been found to be a strong predictor of performance decrement (42, 43), with similar *R*^2^ values found across a wider range of conditions during numerous 20 km (*R*^2^ = 0.77) and 50 km (*R*^2^ = 0.70) racewalk events (44). It may be that pace slowed over time because the requirement for cutaneous blood flow results in a reduction in skeletal muscle blood flow, ultimately inhibiting the ability to continue exercise at the same intensity (45). T_sk_ is pertinent because at high cutaneous blood flow, central blood volume is compromised causing a reduction in stroke volume, which cannot be compensated for by heart rate, causing a decline in cardiac output and degradation in V̇o_2max_ as delivery to the skeletal muscle is reduced, augmenting fatigue (46, 47). In addition, the concomitant cardiovascular strain is a limiting factor (48), with recent research from Foster et al. (32), pointing to a more prominent role for T_sk_ than previously suggested. This was perhaps supported by the best relative performance in the men’s marathon, corresponding to the lowest T_sk_, explained in turn by the less severe environmental conditions compared with the other races. Of note, races with the worst relative performance (men’s 20 km and men’s 50 km) were also those who displayed the greatest rise in MBT. Although this shows the greatest rise in MBT at the end of races, it remained relatively stable throughout and therefore may only be a function of the sharp rise in T_c_ at the end of races, driven by an end-sprint. The results of this study cannot definitively state what physiological factor drove performance, it would therefore be pertinent for future work, to determine which physiological phenomenon is the primary driver.

### Limitations

Collecting data during real-world competition inevitability cannot be controlled to the same degree as a laboratory study. There was no control of athletes’ routines before the race, with clear differences in the use of pre and per-cooling and hydration (12). Gastrointestinal telemetry pills for measuring T_c_ offer a valuable tool, however, the exact location of the pill within the digestive tract cannot be fully controlled. To best account for this, athletes were asked to take pills within 4–6 h before the race. However, many data sets were compromised by incomplete or erroneous data, likely due to either the location of the pill or ingestion of cold fluids resulting in low values. These participants’ T_c_ results were omitted from analysis. Acclimatization status of athletes could not be standardized, with 63% performing between 5 and 30 days of heat acclimation before the event.

For the analysis of T_sk_ emissivity was set to 0.91 for the MWIR and 0.98 for the LWIR. This applies to dry skin within the respective wavelengths, however during activity sweat rate increases and consequently skin wettedness. A layer of sweat on the skin’s surface may act as a filter for infrared (49), the outer layer of which would be measured by the camera. As discussed by Aylwin et al. (8) this is plausible for the LWIR, but not for the MWIR camera where water is more transparent for IR at the respective wavelength. Individual thermograms displayed detailed spatial variations in T_sk_, showing expected mottling. Thus, despite possible measurement of the thin water layer, the camera is still highly sensitive to regional variations in T_sk_. Importantly, for heat transfer purposes, it is the temperature of the thin water layer that determines dry and evaporative heat loss, as it determines the surface vapor pressure. For the MWIR it may be assumed, for thin sweat layers, that the value observed in the present data is likely a combination of T_sk_ and sweat temperature, whereas for the LWIR it will be the surface, as discussed by Aylwin et al. (8). Given the similar outcomes for both cameras (8) the thin sweat layer during the activity does not seem to have had much impact.

### Conclusions

In conclusion, high levels of thermal strain were experienced by athletes in the out-of-stadium events at the 2019 IAAF World Athletics Championships, under hot-humid, night-time conditions. This study provides novel indications of the temporal pattern of thermoregulatory strain, along with performance during elite competition, utilizing both high quality T_sk_ measurement and continuous T_c_ measurement together. Contrary to earlier laboratory findings, T_sk_ declined during races, suggesting a greater need for field-based research or more representative conditions in laboratory testing. Lower T_sk_ was seen in races with lower environmental conditions and external cooling. T_c_ increased initially more rapidly, then slowed and displayed a rapid rise again toward the end of races, mirroring pacing profiles. As this did not correlate with performance it seems likely that T_c_ is an outcome of performance rather than a driver. However, there was a strong relationship between WBGT and performance, with greater degradation in performance seen with the greatest T_sk_ and therefore MBT. Some athletes displayed T_sk_ indicative of aggressive cooling both prerace and in-race but saw no advantage in maintaining a lower T_c_ than other athletes. Finally, the data show that interrupting exercise briefly to take IRT measurements may lead to higher values compared with IRT measurements during the activity.

## DATA AVAILABILITY

Data will be made available upon reasonable request.

## GRANTS

This work received funding support from World Athletics (formerly IAAF).

## DISCLOSURES

No conflicts of interest, financial or otherwise, are declared by the authors.

## AUTHOR CONTRIBUTIONS

G.H., M.C., A.L., M.I., L.T., P.E.A., M.A., J.-M.A., N.B., S.B., C.E., J.G.-E., F.G., M.L., G.L., S.M., K.M., N.T., M.W., S.B., and S.R. conceived and designed research; G.H., M.C., A.L., M.I., L.T., P.E.A., M.A., J.-M.A., N.B., S.B., C.E., J.G.-E., F.G., M.L., G.L., S.M., K.M., N.T., M.W., S.B., and S.R. performed experiments; P.A. analyzed data; P.A. interpreted results of experiments; P.A. prepared figures; P.A. drafted manuscript; P.A., G.H., M.C., M.I., L.T., P.E.A., M.A., J.-M.A., N.B., S.B., C.E., J.G.-E., F.G., M.L., G.L., S.M., K.M., N.T., M.W., S.B., and S.R., edited and revised manuscript; P.A., G.H., M.C., A.L., M.I., L.T., P.E.A., M.A., J.-M.A., N.B., S.B., C.E., J.G.-E., F.G., M.L., G.L., S.M., K.M., N.T., M.W., S.B., and S.R. approved final version of manuscript.

## Figures and Tables

**Figure A1.**
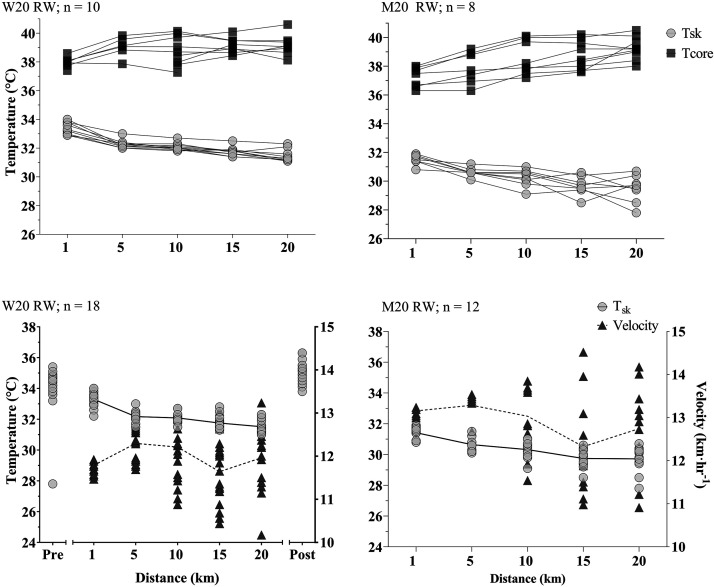
Individual core (T_core_) and weighted mean skin temperature (T_sk_) and velocity during the Women’s (W) and Men’s (M) 20 km racewalks.

**Figure A2.**
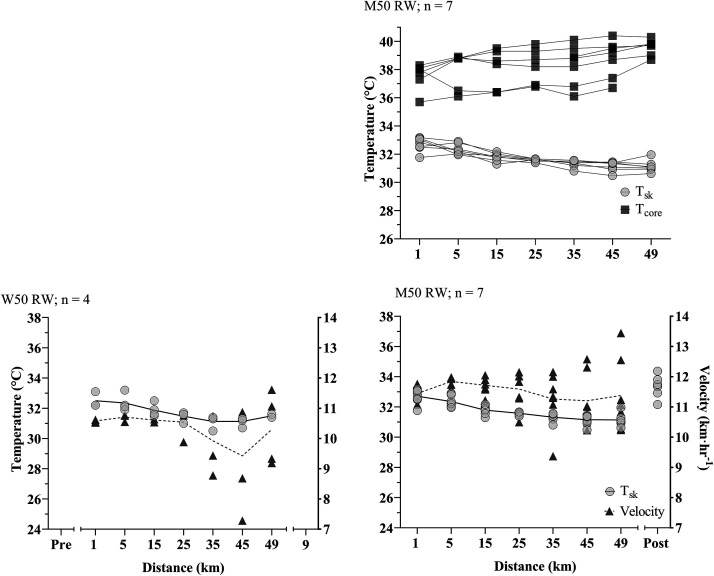
Individual core (T_core_) and weighted mean skin temperature (T_sk_) and velocity during the Women’s (W) and Men’s (M) 50 km racewalks.

**Figure A3.**
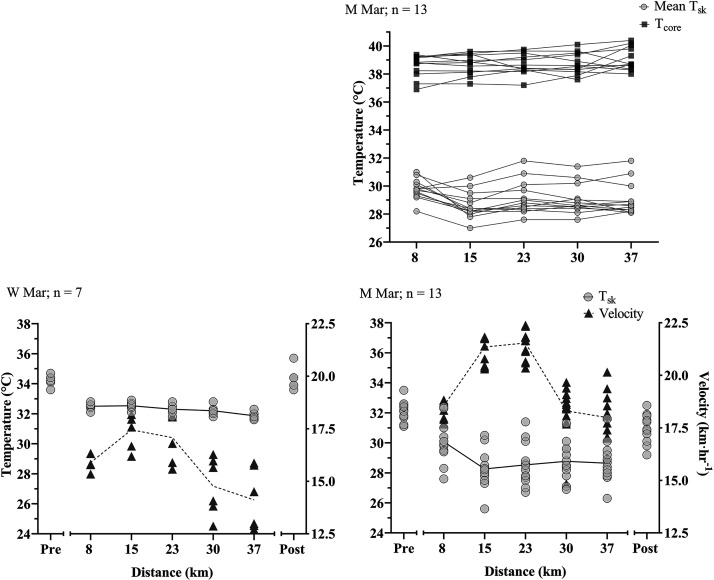
Individual core (T_core_) and weighted mean skin temperature (T_sk_) and velocity during the Women’s (W) and Men’s (M) marathons.

## APPENDIX

Details on the weighting factors used to calculate the mean exposed skin temperature from the different analyzed body skin sections are provided in Table A1 for females and in Table A2 for the males. This is followed by the data presented in the main paper, but now showing individual data rather than means and standard deviations. To allow a better judgment by the reader of the variety in individual responses. These data are shown in Fig. A1 for the women and men’s 20 km racewalks, in Fig. A2 for the women and men’s 50 km racewalks, and finally in Fig. A3 for the women and men’s marathons.

**Table A1.** Weighting of each body site for mean exposed T_sk_ calculation, for the frontal (anterior), lateral, and rear (posterior) aspects, for females

	Anterior	Lateral	Posterior
Face	0.081	0.043	
Neck	0.037		
Torso	0.157	0.076	0.098
Hands		0.025	
Shoulders	0.063	0.039	0.103
Upper arm	0.073	0.092	0.106
Forearm	0.038	0.073	0.063
Thigh	0.349	0.399	0.388
Calf	0.202	0.253	0.243
Total	1	1	1

T_sk_, skin temperature.

**Table A2.** Weighting of each body site for mean T_sk_ calculation, for the frontal (anterior), lateral, and rear (posterior) for males

	Anterior	Lateral	Posterior
Face	0.096	0.047	
Neck	0.044		
Torso			
Hands		0.027	
Shoulders	0.075	0.042	0.114
Upper arm	0.086	0.010	0.117
Forearm	0.046	0.079	0.069
Thigh	0.414	0.432	0.430
Calf	0.239	0.273	0.270
Total	1	1	1

T_sk_, skin temperature.
